# Sebnif: An Integrated Bioinformatics Pipeline for the Identification of Novel Large Intergenic Noncoding RNAs (lincRNAs) - Application in Human Skeletal Muscle Cells

**DOI:** 10.1371/journal.pone.0084500

**Published:** 2014-01-06

**Authors:** Kun Sun, Yu Zhao, Huating Wang, Hao Sun

**Affiliations:** 1 Department of Chemical Pathology, Li Ka Shing Institute of Health Sciences, Prince of Wales Hospital, The Chinese University of Hong Kong, Hong Kong SAR, China; 2 Department of Obstetrics and Gynaecology, Li Ka Shing Institute of Health Sciences, Prince of Wales Hospital, The Chinese University of Hong Kong, Hong Kong SAR, China; Huazhong University of Science and Technology, China

## Abstract

*Ab initio* assembly of transcriptome sequencing data has been widely used to identify large intergenic non-coding RNAs (lincRNAs), a novel class of gene regulators involved in many biological processes. To differentiate real lincRNA transcripts from thousands of assembly artifacts, a series of filtering steps such as filters of transcript length, expression level and coding potential, need to be applied. However, an easy-to-use and publicly available bioinformatics pipeline that integrates these filters is not yet available. Hence, we implemented sebnif, an integrative bioinformatics pipeline to facilitate the discovery of *bona fide* novel lincRNAs that are suitable for further functional characterization. Specifically, sebnif is the only pipeline that implements an algorithm for identifying high-quality single-exonic lincRNAs that were often omitted in many studies. To demonstrate the usage of sebnif, we applied it on a real biological RNA-seq dataset from Human Skeletal Muscle Cells (HSkMC) and built a novel lincRNA catalog containing 917 highly reliable lincRNAs. Sebnif is available at http://sunlab.lihs.cuhk.edu.hk/sebnif/.

## Introduction

Recent advances in transcriptome sequencing have led to the identification of many lincRNA transcripts (>200 nucleotides) [1], [2], [3] that localize in the intergenic region of protein coding genes (mRNAs). These transcripts have very weak or no coding potential for any protein products; their expression levels are generally lower than that of mRNAs thus are often mistakenly considered as transcriptional noises; many of them are transcribed by Polymerase II (Pol II) and spliced like mRNAs while a significant portion of them remain as single-exonic transcripts [3], [4]. Emerging evidence suggests that lincRNAs are functional transcripts in various biological systems under different physiological and pathological conditions. In addition, the number of lincRNAs in mammalian species is estimated to be at least twice the number of mRNAs [5] with the majority of them are still undiscovered. Therefore, fervent efforts are being made in identifying novel lincRNAs in various biological systems.

Whole genome transcriptome sequencing, also known as RNA-seq, coupled with *ab initio* assembly has become an effective approach to discover novel lincRNAs [6]. To this end, RNAs are converted to cDNAs and subjected to high throughput sequencing; the obtained raw reads are then aligned to a reference genome and compared to known gene annotations to generate a list of novel transcripts. However, a high portion of the assembled transcripts are artifacts from genomic contamination or alignment bias, which could be falsely identified as novel lincRNAs. Therefore, the key issue is how to discriminate *bona fide* novel lincRNA transcripts from thousands of assembly artifacts. A widely used approach is to apply several filters, such as filters of transcript length, expression level and coding potential, to remove these artifacts step by step [1], [7], [8]. This multi-filtering approach has been proven effective in discovering thousands of novel multi-exonic lincRNAs in various systems [1], [7], [8], [9]. But a large number of single-exonic transcripts were often discarded simply due to the lack of effective ways to discriminate them from thousands of the assembled artifacts. On the other hand, more and more studies have demonstrated that single-exonic lincRNAs are indeed functional. Well-characterized examples include MALAT1 [10], NEAT1 [11], Xist [12], HOTAIR [13] and Yam-1 [14]. Therefore, single-exonic transcripts should be considered as an important subclass in lincRNA families; and algorithms towards identification of unknown single-exonic lincRNA transcripts need to be developed. Furthermore, a bioinformatics pipeline, which integrates these filtering steps, is not yet publicly available. To fill these gaps, we designed and implemented an integrative bioinformatics pipeline named sebnif (Self-Estimation Based Novel LincRNA Filtering pipeline) to facilitate the identification of both multi- and single-exonic lincRNAs. To illustrate its usage and performance, we applied it on a RNA-seq dataset from Human Skeletal Muscle Cells (HSkMC) to build a lincRNA catalog. Further analysis of these novel lincRNAs reveals their specific genomic distribution pattern and potential functions.

## Methods

### Pipeline overview

**Figure 1:**
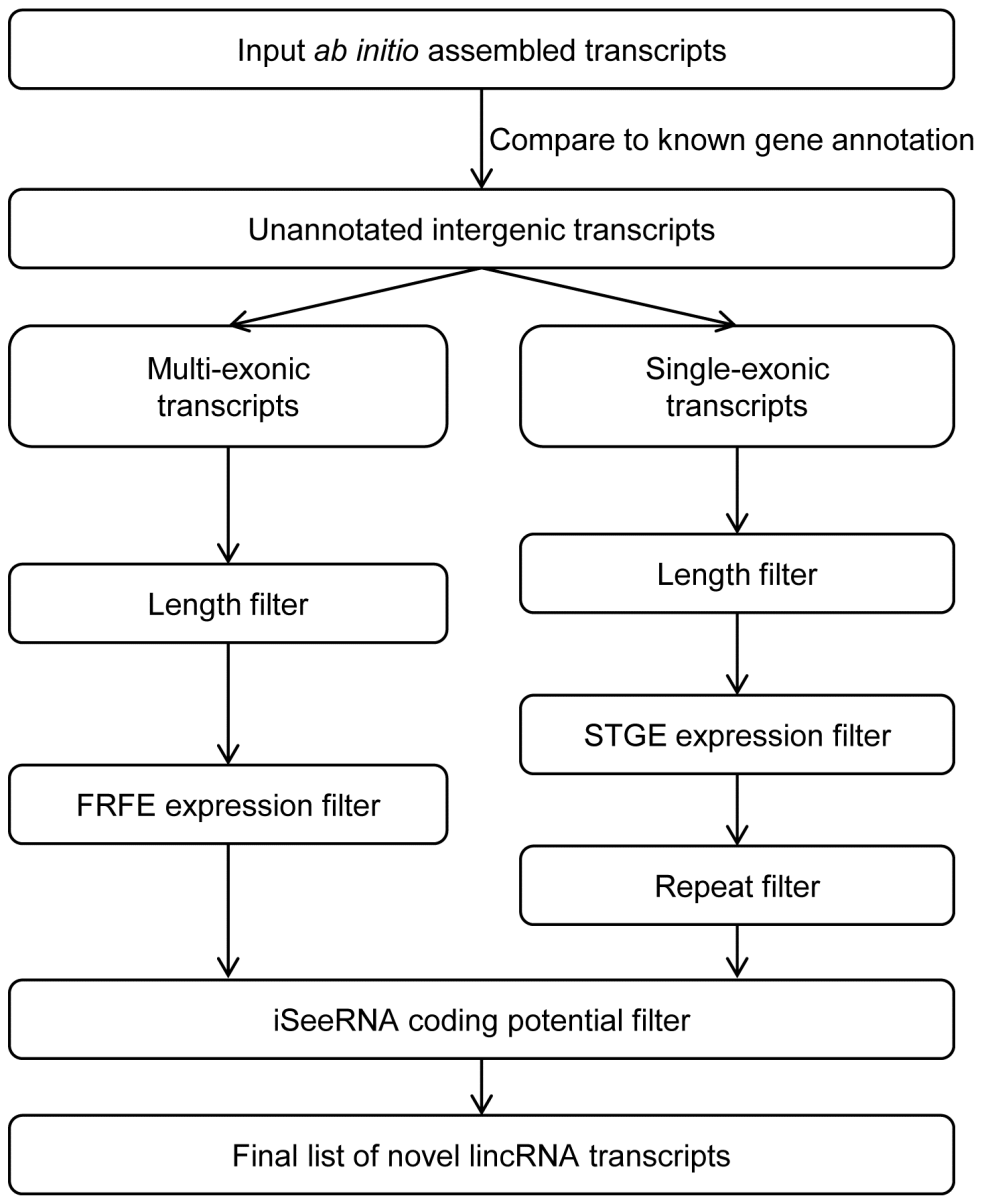
Schematic overview of sebnif. Key steps and filters are illustrated. FRFE: Fully Reconstruction Fraction Estimation; STGE: Single-exonic Transcript Gaussian/Gamma Estimation.

Sebnif is a comprehensive pipeline for the identification of novel lincRNAs from transcriptome sequencing data. It integrates several important filtering and annotation steps to eliminate the assembly noise and enhance the quality of identified lincRNAs. In practice, it provides an array of options (with optimized default values, Table 1) to offer great flexibility for analyzing data according to the given biological question. The typical workflow is depicted in Figure 1 and elaborated as follows:

**Table 1:** Description of the parameters of sebnif and their default values.

Parameter	Description	Default value
-g	Specify species	Homo
-r	Specify reference annotation	RefSeq
-m	Set length cutoff for multi-exonic transcripts	200
-s	Set length cutoff for single-exonic transcripts	200,10000
-F	Set FRFE cutoff	0.5^a^
-E	Set STGE cutoff	0.05,0.95
-X	Specify the model used in STGE	auto^b^
-p	Set repeat region filter cutoff	0.05
-n	Set iSeeRNA noncoding score cutoff	0.9
-o	Set output directory	sebnif

a. Set ‘-F auto’ will command sebnif to use the overall balanced threshold.

b. Set ‘-X auto’ will command sebnif to determine the appropriate model.

### Input files

As a downstream pipeline designed to filter assembled transcripts, sebnif uses the outputs of commonly used assembly software, Cufflinks [15] and Scripture [9]. No special settings are required during the assembly procedure, but it must be in “*ab initio*” mode when using Cufflinks (both ‘-g’ and ‘-G’ options are not specified; for more information please refer to the Cufflinks manual at http://cufflinks.cbcb.umd.edu/manual.html) or in “segmentation task” mode for Scripture (see Scripture manual at http://www.broadinstitute.org/software/scripture/Segmentation_task). As input, it accepts a General Feature Format (GFF) or Gene Transfer Format (GTF) which is one of the widely accepted standard file formats used to store the transcript structure and associated annotation information. Sebnif can use the output of Cufflinks directly since it is in GFF/GTF format; for Scripture, which outputs files in Browser Extensible Data (BED) format, sebnif implements a utility program to convert it to GFF/GTF format. The detailed information on these two file formats can be found at UCSC genome browser (http://genome.ucsc.edu/FAQ/FAQformat.html).

### Implementation of filtering steps

Subsequently, we implement multiple filtering steps as key components of sebnif (Figure. 1):

#### Step1: Filter of annotated known transcripts

To eliminate annotated known transcripts (both protein coding and noncoding), sebnif first compares the *ab initio* assemblies with reference known gene annotations (Table 1, “-g” and “-r” options). Currently, sebnif provides annotations from: (1) RefSeq [16] for Homo Sapiens (NCBI37/UCSC hg19) and Mus Musculus (NCBI37/UCSC mm9); and (2) GENCODE [17] for Homo Sapiens (NCBI37/UCSC hg19) only. Meanwhile, sebnif also allows the users to provide their own gene annotation if available. During the comparison, sebnif eliminates those assembled transcripts with at least one exon overlapping with any annotated known transcript and those falling into the intron regions. The remaining unannotated intergenic transcripts are collected and split into either multi- or single-exonic group which will then be processed separately in the pipeline (Figure 1).

#### Step2: Filter of transcript length

Next, sebnif, by default, filters the transcripts whose lengths are shorter than 200 bp or unreasonably long (e.g. >10 kbp) resulting from the assembly artifacts or un-spliced pre-mRNAs. However, to provide more flexibility, sebnif provides the option for users to set the lower and upper thresholds based on their requirements. Considering the length distributions of single- and multi-exonic transcripts are normally different (multi-exonic transcripts are generally longer than single-exonic ones [4]), sebnif also allows the users to set different length thresholds for them (Table 1, “-m” and “-s” options).

#### Step3: Filter of the transcript expression level

As many lincRNAs are expressed at a much lower level than the majority of mRNAs, it is very challenging to find an optimal expression level threshold to differentiate lowly expressed *bona fide* lincRNA transcripts from the assembly artifacts. To overcome this challenge is the key focus of sebnif. Considering the structure of the multi- and single-exonic transcripts are very different (single-exonic transcripts do not have exon-intron chain), sebnif implements two separate algorithms to identify the optimal thresholds. For multi-exonic transcripts, sebnif implements a Fully Reconstruction Fraction Estimation (FRFE) approach originally described by Guttman et al. [9]. Briefly, we first divide the multi-exonic transcripts in reference annotation into N expression quantiles based on their expression values. At each expression quantile, we then divide the reference transcript set into two categories based on the assembly results: (1) fully reconstructed where the assembled transcripts capture the exact exon-intron chain as the reference annotation; and (2) otherwise partially reconstructed transcripts. At each expression quantile, the assembly quality can be evaluated by the Fully Reconstruction Fraction (FRF) value which is defined by the proportion of the fully reconstructed transcripts. Based on the FRF value at each expression quantile, sebnif determines the optimal FRFE threshold by balancing the sensitivity and specificity as described in Sun *et al.* [18]: using the fully reconstructed transcripts as positive data set and partially constructed transcripts as negative dataset, at each expression quantile *i*, the sensitivity (*sens[i]*) and the specificity (*spec[i]*) for that quantile can be calculated through the proportion of fully and partially reconstructed transcripts. The overall balanced FRFE threshold corresponds to the lower boundary of the expression level of the quantile *i* that is obtained from minimizing *e_i_* in the following equation:
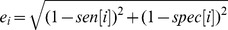



Where *i* belongs to [1, N] and *e_i_* is a measurement of balanced sensitivity and specificity [18].

**Figure 2:**
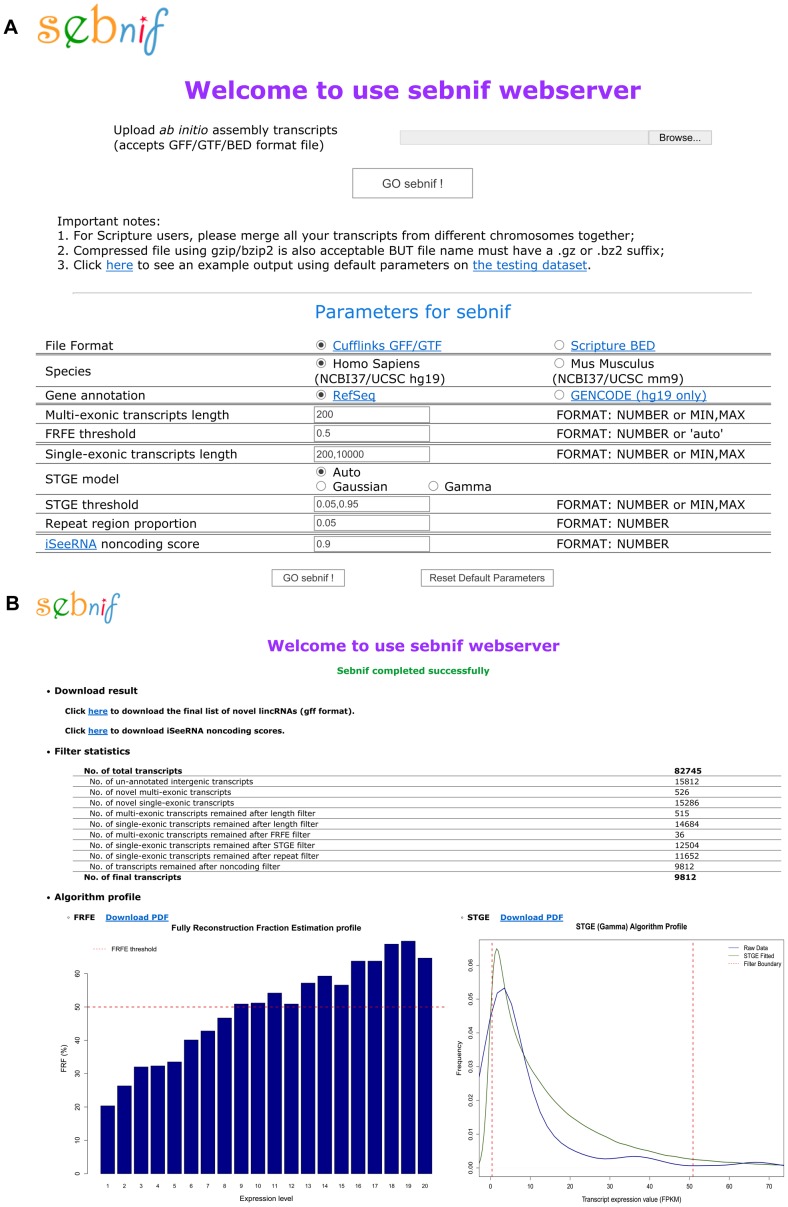
Snapshots of sebnif web server. (A) The data upload page. All the parameters of sebnif could be specified by the users through this page. (B) The result page showing the report of novel lincRNAs identified in Human Skeletal Muscle Cells. The final list of novel lincRNAs in standard GFF format and the iSeeRNA noncoding score for each transcript can be downloaded directly; statistic numbers during the filtering steps and the FRFE Profile and STGE Profile generated by FRFE and STGE algorithms were also provided for users to evaluate the quality of the data.

Besides using this overall balanced FRFE threshold, sebnif also provides an option for users to determine the FRFE cutoff based on the quality of their data (i.e. sequencing depth and performance of the aligner and assembler as well as their needs of stringency) (Table 1, “-F” option). Only those with expression level higher than the threshold corresponding to the user defined FRFE cutoff will be kept. For instance, a 0.5 FRFE threshold suggests that for each transcript that passed the FRFE filter, it should have at least 50% probability to be fully reconstructed (Figure 2B).

Since single-exonic transcripts do not have exon-intron chain, it is impossible to apply FRFE algorithm on them. Sebnif thus implements a novel approach, named Single-exonic Transcript Gaussian/Gamma Estimation (STGE), to estimate the optimal expression threshold. This is based on the fact that the logarithmic gene expression values often follow a Gaussian distribution (also known as normal distribution) pattern [19] or a Gamma distribution [20]. In the STGE algorithm, sebnif first determines the appropriate model by fitting the expression values of the single-exonic transcripts in the reference gene annotation. Then based on the fitted model, a transcript whose expression falls into either tail of the distribution is considered unreliable and discarded. Users have an option to select the model in STGE algorithm or leave it for sebnif to determine (Table 1, ‘-X’ option); and the probability cutoffs used by the filter can also be decided by the users through the options provided by sebnif (Table 1, “-E” option). In our implementation, not only the lower bound but also the upper threshold could be set independently since the transcripts with abnormally high expression may not be reliable lincRNAs; they could result from ribosomal RNAs contamination, potential pseudo-gene, pre-miRNA or alignment bias towards specific regions (e.g. repeat regions as discussed below).

#### Step4: Filter of repeat regions

Due to the sequence similarity, large-scale transcriptome analysis is often biased against repeat elements [21]. This bias has a significant effect on the results of the *ab initio* assembly software especially for the single-exonic transcripts because many individual reads originally from these regions may sometimes be mapped to multiple positions in the genome when not using paired-end RNA sequencing. To minimize this bias, sebnif filters out single-exonic transcripts that contain high percentage of repeat sequences. This percentage threshold can be set up with the option provided for the users (Table 1, ‘-p’ option).

#### Step5: Filter of coding potential

Finally, to eliminate those transcripts with high coding potential (i.e. potential protein coding transcripts), sebnif employs our recently developed software, iSeeRNA [22]. For each transcript, iSeeRNA reports whether it is a coding or non-coding transcript with a non-coding score reflecting the confidence of the prediction. Sebnif filters those transcripts with an iSeeRNA non-coding score lower than the user-defined threshold (Table 1, “-n” option).

### Further annotation of novel lincRNAs

To further increase the confidence of the lincRNAs identified from the above steps, sebnif provides several utility programs to annotate each lincRNA with genomic features around its promoter and gene body, such as tri-methylation of lysine 4, and 36 of histone H3 (H3K4me3, and H3K36me3), Expressed Sequence Tag (EST) and Cap Analysis of Gene Expression (CAGE) tags; these features are thought to be generally associated with active or expressed transcripts. The utility programs are compatible with several widely used file formats such as BED, SAM (Sequence Alignment/Map), BAM (compressed binary version of SAM) and GFF/GTF. Additionally, the outputs can be easily submitted to further filtering steps.

### Implementation and availability

Sebnif is implemented in Perl (http://www.perl.org/) and R [23], [24] and runs on most Unix/Linux machines. Source code package is freely available at http://sunlab.lihs.cuhk.edu.hk/sebnif/ and distributed under the Boost Software License (http://www.boost.org/LICENSE_1_0.txt). Sebnif is easy to install and only depends on iSeeRNA, which has been included in the package. The package also contains a testing dataset, which has ∼80,000 *ab initio* assembled transcripts from Human Skeletal Muscle Cells (HSkMC) RNA-seq data. To demonstrate how to use sebnif, a shell script is provided to integrate the data analysis workflow by first configuring iSeeRNA and then running on the testing dataset. Meanwhile, to facilitate the usage of sebnif, especially by those wet lab biologists with minimal informatics background, we also implement sebnif as a user-friendly web server with free access at http://sunlab.lihs.cuhk.edu.hk/sebnif/webserver/ (Figure 2A, 2B). Like the stand-alone package, the web server currently supports two species: Homo Sapiens (UCSC hg19) and Mus Musculus (UCSC mm9). Users can upload their *ab initio* assembled transcripts in GFF/GTF or BED format. All the parameters of sebnif can be specified by the users through the web interface (Figure 2A).

### Output files

The main output files of sebnif include a standard GFF file containing a list of novel lincRNA transcripts as well as a text file with non-coding scores calculated by iSeeRNA. If using sebnif web server, both files can be downloaded from the output webpage (Figure 2B). In addition, a text file with important statistics (i.e. the number of transcripts that passed or failed each filter) and two figures illustrating the expression filter thresholds of FRFE and STGE algorithms are also included (Figure 2B). We aim to provide not only the list of identified novel lincRNA loci but also adequate information for users to evaluate the quality of their data and the performance of the filtering procedures.

### Novel lincRNA validation by RT-PCR

To validate the identified novel lincRNA transcripts from HSkMC, we used RNAs extracted from human skeletal myoblasts (Life Technology). In summary, total RNAs were extracted using Trizol reagent (Invitrogen) according to the manufacturer's protocol and treated with DNase I (1 U/ µl; Invitrogen) to remove possible contaminating genomic DNA. cDNAs were then reverse transcribed from DNA-free RNAs using M-MLV Reverse Transcriptase (Invitrogen) and diluted 5-fold for further PCR analysis. For validation of the target transcripts, primer sets were designed using web-based tool Primer-Blast (http://ncbi.nlm.nih.gov/tools/primer-blast) and specificity was checked with human RefSeq mRNA. Semi-quantitative RT-PCR was performed using ABI Prism 7900HT Sequence Detection System in a total volume of 5 ul in each well containing 2.5 ul of Power SYBR Green PCR Master Mix (Applied Biosystems), 1 ul cDNA, 1 ul primers (2uM each) and 0.5 ul nuclease-free water. Amplification conditions were as follows: 2 min at 50°C, 10 min at 95°C followed by 35 cycles of 15 s at 95°C and 1 min at 60°C. After amplification, PCR products were separated by electrophoresis on 1.5% agrose gels.

## Results

### Computational requirements of sebnif

To measure the computational requirements of sebnif, we investigated the running time and the memory usage by applying it on the testing dataset, a typical Cufflinks *ab initio* assembly output which contains around 80,000 assembled transcripts. On our Linux server using an Intel Xeon X5675 CPU with single thread, sebnif finished the data processing within five minutes. The peak memory usage was less than 600 MB. These results indicate that sebnif is suitable to run on most of the desktop computers.

### Identification of novel lincRNAs in HSkMC

**Figure 3:**
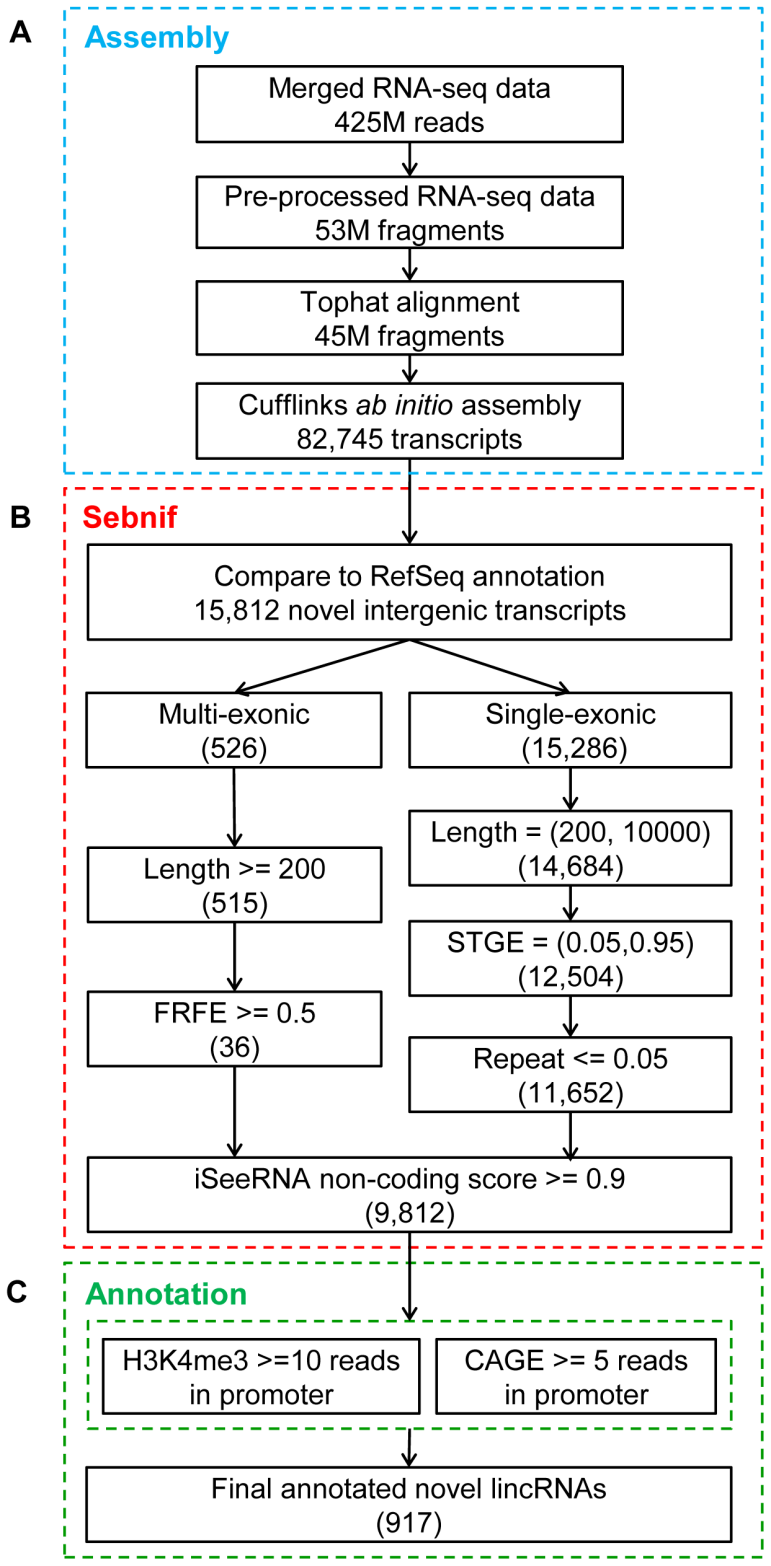
Identification of novel lincRNA catalog in HSkMC. (A) The raw RNA-seq data was pre-processed, aligned with Tophat and assembled using Cufflinks in *ab initio* mode. (B) Sebnif filtering on the assembled transcripts. The numbers in parentheses represent the number of transcripts after each filtering step. (C) Annotating and further filtering of the novel lincRNAs with H3K4me3 and CAGE data.

To illustrate the usage and performance of sebnif on real biological data, we applied sebnif on a publicly available RNA-seq dataset from HSkMC. Raw RNA-seq data (FASTQ reads) was downloaded from the Encyclopedia of DNA Elements (ENCODE) project [25]. This dataset comprises a total of ∼425 million paired-end reads from two samples sequenced by Illumina Hi-seq 2000. After downloading the sequenced fragments, we first trimmed the adapters, removed the duplicated reads (pre-processing) using in-house programs, and then aligned the remaining fragments to the reference human genome (hg19) using Tophat (version 2.0.6) [26] guided by the GENCODE gene annotation (version 16) (the ‘-G’ option). We performed the *ab initio* assembly on the aligned fragments using Cufflinks (version 2.1.1) (Figure 3A) using all the default parameters to generate 82,745 transcripts, which were then processed through sebnif with all the default parameters (Table 1) (For more details, please refer to the README file of sebnif at http://sunlab.lihs.cuhk.edu.hk/sebnif/README). As aforementioned, sebnif applied multiple filters on these transcripts (Figure 3B). First, it removed the assemblies overlapping with transcripts annotated in RefSeq. This led to the removal of more than 80% assembled transcripts that are either reconstructed protein coding transcripts or annotated known non-coding transcripts. Since we are interested in the non-coding transcripts in intergenic regions, it also removed those transcripts partially overlapping with exons or introns of the annotated known transcripts (Figure S1). This filter resulted in the identification of 15,812 novel intergenic transcripts (Figure. 3B), among which 526 and 15,286 are multi- and single-exonic transcripts respectively. For both categories, those shorter than 200 were removed; single exonic transcripts longer than 10,000 nucleotides were also removed. After passing this length filter, 515 multi- and 14,684 single-exonic transcripts remained. Next, to remove the unreliable assembled transcripts with extremely low expression level, we applied the expression filter on 15,199 novel transcripts. For 515 multi-exonic transcripts, sebnif applied a FRFE threshold of 0.5. For single-exonic transcripts, sebnif used STGE to model the transcript expression profiling and selected the expression levels corresponding to the probabilities of 0.05 and 0.95 as lower and upper probability cutoffs. With these cutoffs, single-exonic transcripts with too low (below 5%) or too high (above 95%) expression level were eliminated. The final numbers of transcript after these filters are 36 and 12,504 transcripts for multi- and single-exonic respectively. When applying repeat region filter to remove single-exonic transcripts that have more than 5% of its sequence overlapping with the repeat regions, 6.8% (852 of 12,504) failed to pass this filter, leaving a total number of 11,625 transcripts. Next, iSeeRNA was used to calculate the non-coding score for each transcript and those with a non-coding score lower than 0.9 (i.e. potential protein coding transcripts) were removed. Finally 9,812 novel transcripts were obtained as a provisional catalog of lincRNA loci in HSkMC (Dataset S1).

### Annotating of novel lincRNA loci with genomic features associated with transcriptional activation

To further annotate our novel lincRNA transcripts and gain more confidence of the above filtering, a total of 13.3 and 55.8 million aligned reads from H3K4me3 ChIP-seq experiment and CAGE tags were downloaded from ENCODE project and used to annotate each novel lincRNA using the utility program provided by sebnif (Figure 3C). Only those with at least 10 H3K4me3 reads and 5 CAGE tags in their promoter regions (i.e. 2 kbp upstream to 1 kbp downstream of transcript start site (TSS)) were kept. As a result, a total of 917 novel lincRNAs were identified as the final list (Table S1).

### Validation of the novel lincRNAs

**Figure 4:**
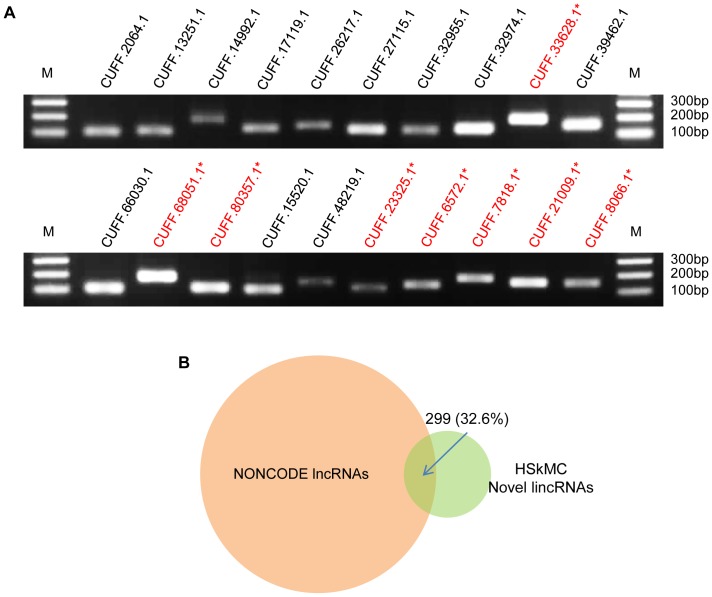
Validation of the novel lincRNAs. (A) 26 randomly selected novel lincRNAs from the final lincRNA list were subjected to RT-PCR validations, among which 8 were divergent lincRNAs (the transcript id is marked in red color and ends with a ‘*’ suffix). The PCR products were visualized on Agoras gel and the sizes of DNA markers (M) are shown on the right. (B) Comparison of the identified novel lincRNAs with NONCODE v3.0. 299 transcripts (32.6%) were found in common.

To validate the identified novel lincRNA loci especially the single-exonic ones, we randomly selected 26 single-exonic transcripts and tested their presence via RT-PCR in human skeletal myoblasts. 20 out of 26 were found to be expressed at a detectable level (Figure 4A and Table S2). The 6 failed transcripts were among the lowest expressed by RNA-seq (Cufflinks FPKM (Fragments Per Kilobase of transcript per Million mapped reads) <1) (Table S2). These results indicate that sebnif can indeed produce high quality list of novel lincRNAs. To further validate our lincRNA findings, we also compared our list with the NONCODE v3.0 database [27] which contains 33,818 human long noncoding RNAs (lncRNAs) collected from the published literatures. 299 (32.6%) were found in NONCODE v3.0 and 6 have the exact same transcript structure (Figure 4B, Table S3), further indicating that sebnif identified lincRNAs are highly likely to be *bona fide* lncRNAs.

### HSkMC lincRNAs are less conserved and lowly expressed

**Figure 5:**
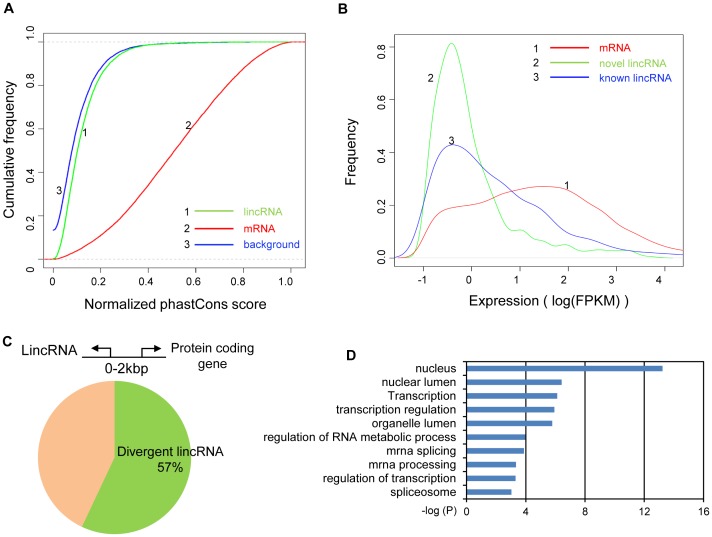
Analysis of the novel lincRNAs in HSkMC. (A) Cumulative curve of the average PhastCons score of the novel lincRNAs (green) compared to randomly selected genome background (blue) and known mRNAs (red). These novel lincRNAs are more conserved than the genome background but less conserved than the mRNAs. (B) Comparison of expression profiles of novel lincRNAs (green), known ncRNAs (blue) and known mRNAs (red). Both novel lincRNAs and the known ncRNAs are expressed at a lower level than known mRNAs. (C) 57% (523 out of 917) of the novel lincRNAs are divergent transcripts generated within 2 kbp upstream of known protein coding genes. (D) Gene Ontology annotation of the above protein coding genes. The y-axis shows the top 10 enriched GO terms and the x-axis shows the enrichment significance P-values.

To study the characteristics of the newly identified HSkMC lincRNAs, we first calculated the conservation scores (PhastCons score [22], [28]) for three types of genomic loci: (1) novel lincRNA loci identified from this study; (2) RefSeq annotated protein coding loci; and (3) randomly selected genomic loci within intergenic regions. When comparing the conservation scores, we found novel lincRNA loci displayed moderate conservation which is lower than that of the protein coding gene loci but slightly higher than that of the randomly selected genomic loci (Figure 5A). When comparing the expression levels, both novel and annotated known lincRNAs displayed similar levels which were generally lower than protein coding mRNAs (Figure 5B). These results are consistent with the findings from previous studies [4], [7], [9], [29].

### Promoter associated lincRNAs

When further inspecting the genomic distribution, we discovered that many (57%) of the HSkMC lincRNAs are originated within 2 kbp upstream region of the TSSs of known protein coding genes (Figure. 5C), in keeping with previous report that a large fraction of lncRNA transcripts are promoter associated transcripts that originate from divergent transcription at promoters of active protein-coding genes [30]. Among the 20 validated lincRNAs, 8 are annotated as promoter associated lincRNAs (Figure. 4A). We further performed Gene Ontology (GO) analysis on those protein coding genes paired with lincRNAs using DAVID [31], [32]. The result shows a strong enrichment of GO terms including “nucleus” and “transcriptional regulation” (Figure 5D and Table S4).

## Discussion

Sebnif is one of the first bioinformatics pipelines designed and implemented as a publicly available web server as well as a stand-alone application to facilitate the novel lincRNA discovery. It implements key filtering steps to eliminate low quality and protein coding transcripts. The most unique feature of sebnif lies in its differential treatment of multi- and single-exonic transcripts. Firstly, it implements FRFE algorithm that allows the users to use FRFE threshold for filtering multi-exonic transcripts. Compared with the commonly used expression level (FPKM value) or read coverage of the transcript [1], [7], FRFE threshold is a direct and consistent measurement of the overall assembly quality across different samples. For example, an expression level of FPKM = 1 may correspond to FRFE threshold of 80% in one dataset but only 60% in another, leading to inconsistency of the overall filtering quality. In addition to employing a balanced FRFE cutoff, it also allows users to set up FRFE value cutoff manually, which is very useful when the sequencing data quality is not ideal. Secondly, sebnif is the first bioinformatics pipeline specifically designed for filtering single-exonic transcripts by implementing the STGE algorithm. Emerging evidence show the existence of a large proportion of functional single-exonic lincRNAs, thus omitting them to simplify the identification pipeline will affect the completeness of the lincRNA catalog. Through the STGE algorithm, a complete catalog of lincRNAs can be obtained which will facilitate the associated functional studies.

To further demonstrate the usage of sebnif, 917 high confidence novel lincRNAs were identified from HSkMC. A large portion of these lincRNAs are single-exonic, again demonstrating the prevalence of single-exonic transcripts in biological systems. It is also interesting to find out that more than half of the identified lincRNAs are divergent lincRNAs originated from the promoter region of protein coding transcript. This finding is in line with several recent studies [30], [33] demonstrating the prevalence of divergent lincRNA/protein coding gene pairs. Functionally these lincRNAs may regulate their neighboring protein coding genes *in cis* by flagging the chromatin region and recruiting regulatory complex through their RNA-protein binding activities. The enrichment of “transcription” related GO terms indicates that these lincRNAs may be involved in transcriptional regulation through interaction with their associated protein coding genes. It will be an interesting direction to explore in the future.

We also noticed that although a significant proportion (32.6%) of our lincRNAs can be found in the NONCODE v3.0 database, there was little overlapping with the list of lincRNAs identified by Hangauer *et al.* [34] from HSkMC (data not shown). This inconsistency may largely due to the fundamental differences in the identification pipelines. For example, merged reads from 23 tissues were used as a starting point in their study such that the lincRNAs specifically expressed in HSkMC sample may be considered lowly expressed across all tissues thus discarded during the filtering; also, the filtering criteria for both single and multi-exonic transcripts are drastically different from ours.

In conclusion, the approach described herein, coupled with available *ab initio* assembly software has the potential to dramatically speed up the identification of novel lincRNAs and represents an important step in the development of high-throughput lincRNA discovery platform. LincRNA catalog obtained through sebnif provides researchers a list of high quality lincRNAs for further experimental validation and functional study.

## Supporting Information

Figure S1 **Overview of the** ***ab initio*** **assembled transcripts in Human Skeletal Muscle Cells (HSkMC).** A large proportion (64.85%) of the assembled transcripts has not been annotated in RefSeq, among which 19.46% are intergenic transcripts.
(PDF)

Dataset S1 **The list of lincRNA loci after sebnif filtering.** The file contains 9,812 transcripts in standard GFF format which records the structure of the transcripts and their expression values (measured by Cufflinks in FPKM).
(GFF)

Table S1 **The final list of novel lincRNAs in HSkMC.**
(XLSX)

Table S2 **RT-PCR validation of newly identified lincRNAs in HSkMC.**
(XLSX)

Table S3 **List of 6 lincRNAs with the same gene structure found in NONCODE database.**
(XLSX)

Table S4 **List of the divergent lincRNAs and the Gene Ontology (GO) annotations of the associated protein coding genes.**
(XLSX)
