# Supplementary figures and images for: Sebnif: An Integrated Bioinformatics Pipeline for the Identification of Novel Large Intergenic Noncoding RNAs (lincRNAs) - Application in Human Skeletal Muscle Cells
Source: PLoS One. 2014 Jan 6;9(1):e84500. doi: 10.1371/journal.pone.0084500 (PMC3882232; doi:10.1371/journal.pone.0084500)

Suppl. Figure S1 Sun *et. al.*

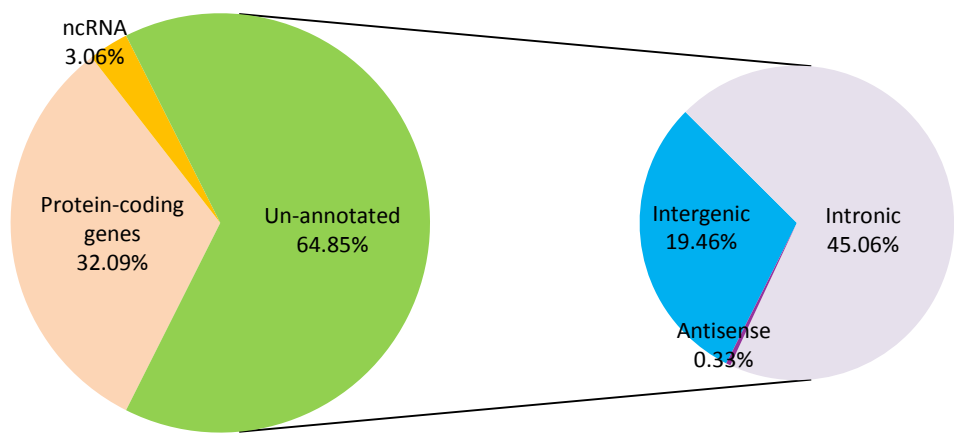

Supplement: Figure S1 — Overview of the ab initio assembled transcripts in Human Skeletal Muscle Cells (HSkMC). A large proportion (64.85%) of the assembled transcripts has not been annotated in RefSeq, among which 19.46% are intergenic transcripts. (PDF) [file pone.0084500.s001.pdf]
